# Bacterial Transformation Buffers Environmental Fluctuations through the Reversible Integration of Mobile Genetic Elements

**DOI:** 10.1128/mBio.02443-19

**Published:** 2020-03-03

**Authors:** Gabriel Carvalho, David Fouchet, Gonché Danesh, Anne-Sophie Godeux, Maria-Halima Laaberki, Dominique Pontier, Xavier Charpentier, Samuel Venner

**Affiliations:** a Université de Lyon, Université Lyon 1, CNRS, Laboratoire de Biométrie et Biologie Evolutive UMR 5558, Villeurbanne, France; b CIRI, Centre International de Recherche en Infectiologie, Inserm, U1111, Université Claude Bernard Lyon 1, Villeurbanne, France; c Université de Lyon, VetAgro Sup, Marcy-l'Étoile, France; d CNRS UMR5308, École Normale Supérieure de Lyon, University of Lyon, Villeurbanne, France; National Center for Biotechnology Information, NLM, NIH; The Ohio State University

**Keywords:** natural transformation, horizontal gene transfer, mobile genetic elements, resistance genes, stochastic environment

## Abstract

Horizontal gene transfer (HGT) promotes the spread of genes within bacterial communities. Among the HGT mechanisms, natural transformation stands out as being encoded by the bacterial core genome. Natural transformation is often viewed as a way to acquire new genes and to generate genetic mixing within bacterial populations. Another recently proposed function is the curing of bacterial genomes of their infectious parasitic mobile genetic elements (MGEs). Here, we propose that these seemingly opposing theoretical points of view can be unified. Although costly for bacterial cells, MGEs can carry functions that are at points in time beneficial to bacteria under stressful conditions (e.g., antibiotic resistance genes). Using computational modeling, we show that, in stochastic environments, an intermediate transformation rate maximizes bacterial fitness by allowing the reversible integration of MGEs carrying resistance genes, although these MGEs are costly for host cell replication. Based on this dual function (MGE acquisition and removal), transformation would be a key mechanism for stabilizing the bacterial genome in the long term, and this would explain its striking conservation.

## Abstract (precis)

Natural transformation is the acquisition, controlled by bacteria, of extracellular DNA and is one of the most common mechanisms of horizontal gene transfer, promoting the spread of resistance genes. However, its evolutionary function remains elusive, and two main roles have been proposed: (i) the new gene acquisition and genetic mixing within bacterial populations and (ii) the removal of infectious parasitic mobile genetic elements (MGEs). While the first one promotes genetic diversification, the other one promotes the removal of foreign DNA and thus genome stability, making these two functions apparently antagonistic. Using a computational model, we show that intermediate transformation rates, commonly observed in bacteria, allow the acquisition then removal of MGEs. The transient acquisition of costly MGEs with resistance genes maximizes bacterial fitness in environments with stochastic stress exposure. Thus, transformation would ensure both a strong dynamic of the bacterial genome in the short term and its long-term stabilization.

## INTRODUCTION

Horizontal gene transfer (HGT), i.e., the passage of heritable genetic material between organisms by means other than parent-offspring transmission, is commonly observed in bacteria (1–3). By promoting the spread of genes of antibiotic or heavy metal resistance and virulence factors, HGT is an important threat to human health (4). Among all known mechanisms by which HGTs occur, one can distinguish HGTs resulting from the infectious and propagative behavior of mobile genetic elements (conjugation and transduction) and HGTs that are exclusively controlled by the bacterial cells (5–7). By far, the most widespread of those is natural transformation, i.e., the import of free extracellular DNA (eDNA) and its integration into the bacterial genome by homologous recombination (8, 9). The DNA import system is expressed under the state of competence, which is triggered by signals that are often elusive and difficult to reproduce under laboratory conditions (10, 11). Despite this difficulty, transformation has been experimentally demonstrated in more than 80 bacterial species distributed throughout the tree of life, indicative of an ancestral origin (8, 10, 12). The list of transformable species keeps growing, now including species that had long been considered incapable of transformation (13, 14). In addition, transformation-specific genes required for the uptake of eDNA (*comEC* and *dprA*) are widespread (see Fig. S1 in the supplemental material) (15; see also reference 16), suggesting that most bacterial species may undergo transformation in their natural habitat.

10.1128/mBio.02443-19.1 FIG S1 Proportion of competence genes in the AnnoTree database. Graphs represent the phylogenic trees of the 23,936 bacterial genomes. Red lines indicate the gene has been found in the genome. (A) *recA* (KEGG identifier K03553), 23,059 genome hits; *recA* is a gene involved in homologous recombination, a core function essential to chromosome maintenance, and is well known for its ubiquity in all bacterial genomes. (B and C) Genes required for transformation, specifically expressed during competence, and are therefore indicators of the ability to perform natural transformation: *comEC* (KEGG identifier K02238), 20,171 genome hits, involved in the uptake of extracellular DNA (B) and *drpA* (KEGG identifier K04096), 21,523 genome hits, involved in protection of the incoming DNA and its integration in the recipient genome (C). DprA acts by directly and specifically interacting with single-stranded DNA (ssDNA) imported through the ComEC channel and then brings to RecA for recombination. Involvement of DprA in processing of ssDNA other than transforming DNA has so far been excluded (generic recombination). (D) In contrast, the distribution of *fliC*, (KEGG identifier K02406), 92,99 genome hits, encoding the subunit of the bacterial flagellum, is consistent with an accessory function. Download FIG S1, PDF file, 0.1 MB. Copyright © 2020 Carvalho et al. 2020 Carvalho et al. This content is distributed under the terms of the Creative Commons Attribution 4.0 International license.

In spite of this ubiquity and that this mechanism has been documented for a long time (17), the evolutionary causes of transformation are complex to disentangle and are still debated. The proposals of the evolutionary function of transformation can be organized into four main categories (see Croucher et al. [18] for a review). First, transformation could be a means of importing eDNA as a nutrient. However, this proposal is questionable, because the competence machinery includes complex and costly mechanisms for the protection or selection of imported DNA (19, 20). Such mechanisms are essential for its successful integration into the genome, whereas they are unnecessary if the imported DNA exclusively serves as a nutrient (10). Second, imported DNA from transformation could be a raw material to repair double-stranded DNA (dsDNA) break. This hypothesis is mainly supported by the fact that the exposition to specific mutagens increases the transformation rate in some species. However, there are also numerous counterexamples of this bacterial response, which calls into question the generic nature of this proposal (18).

Third, the commonly emphasized evolutionary benefits of transformation are the genetic diversification and mixing within bacterial populations, and as a result, transformation is often considered analogous to eukaryotic sexual reproduction. Computational modeling approaches have shown that gene acquisition and genetic mixing from transformation can provide a selective advantage by allowing bacteria to combine favorable mutations (Fisher-Muller effect) (21, 22) and to efficiently exploit new or fluctuating environments (23). Under fluctuating selection of different alleles, transformable bacteria may also benefit from the acquisition of old alleles present in their environment to restore a fitter phenotype (24). In complement to these theoretical investigations, an increasing number of recent studies, in part fueled by the exponential growth of genome sequencing, show that through transformation, bacteria frequently acquire new functions carried by transposons, integrons, and genomic islands (25–28). Transformation enables the acquisition of antibiotic resistance by Campylobacter jejuni and capsule switching by Streptococcus pneumoniae, leading to vaccine escape (29–31). Bacillus subtilis presents a large accessory genome, a diversity seemingly generated by transformation, allowing this species to colonize various ecological niches, from soils and plants to animals (27). Overall, the eDNA obtained from transformation may provide habitat-specific genes and favors adaptation to new environments (32). Yet, genome-based evidences of the benefit of transformation are inherently biased, as they tend to highlight HGT events that result in the acquisition of genes providing clear selective advantage (e.g., antibiotic resistance).

Fourth, in opposition to the genetic diversification and mixing paradigm, Croucher et al. (18) recently proposed the radically distinct hypothesis that the main evolutionary function of transformation is to cure bacterial genomes of integrated genetic parasites, such as bacteriophages. Based on the observation that bacterial genomes are inevitably parasitized by mobile genetic elements (MGEs) (33, 34), they argue that transformation favors the insertion of short DNA sequences from kin cells, thus tending to remove infesting DNA rather than acquire additional foreign DNA (18, 35). Following this “chromosomal curing” hypothesis (18), transformation would be mainly a defense mechanism against infectious genetic parasites rather than a mechanism to acquire new genes or generate genetic diversity and mixing, which would be marginal events. In favor of this proposal, several observations point out that cells are much more likely to undergo recombination with closely related neighbors than with distant lineages carrying foreign DNA (35, 36). Bacteria usually grow close to their siblings, and the emergent physical vicinity favors the exchange of DNA sequences between closely related genotypes. In addition, bacteria may regulate the competence state using quorum sensing, hence ensuring that a critical amount of kin cells are nearby (37). They may also select DNA with specific uptake sequences to avoid integrating exogenous DNA (20). In few cases, competent bacteria kill their own kin cells, enriching their surrounding with clonal DNA (38). Considering all the barriers to the acquisition of foreign DNA, transformation would mainly act as a conservative mechanism instead of a means of genetic diversification and mixing (36).

The third (genetic diversification) and fourth (chromosomal curing) sets of proposals for transformation function are both relevant and supported by solid empirical and simulated data, but they also seem antagonistic. In this theoretical work, we examine the possibility of unifying these two proposals within the same framework by considering the dynamics of MGEs and bacterial populations in fluctuating environments. Unlike the study by Croucher et al. (18), (i) we consider the frequent case of noninfectious MGEs (such as genomic islands) which can integrate into bacterial chromosomes and carry resistance genes but which are also costly for bacteria by reducing their growth capacity, and (ii) we take into account the fact that bacteria evolve in a stochastic environment characterized by unpredictable exposures to stress (e.g., antibiotics and heavy metals). Using a computational model introducing competition between bacterial genotypes with different transformation strategies (transformation rate), we assess whether an optimal transformation rate emerges from the dual function of acquiring then eliminating MGEs carrying resistance genes.

## RESULTS

### Transformable cells are competitive in stochastic environments.

**FIG 1:**
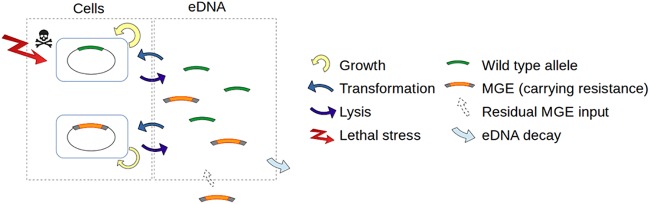
Schematic representation of the computational model. Bacterial cell growth follows a logistic growth model. The bacterial cells have an insertion site in their chromosome at which two types of alleles from the eDNA compartment can be integrated from transformation and replace their current DNA: wild-type (WT) allele and MGE. The integration of a WT allele is costless for cells, whereas the integration of MGEs causes a cost in terms of cell replication. Bacterial populations are faced with stochastic stresses of random duration and intensity. In the absence of stress, cells are lysed at a basal rate. Under stress exposure, the lysis rate of WT cells increases but remains unchanged for cells with an MGE carrying resistance. Each lysed cell releases its DNA and fuels the extracellular compartment with eDNA. MGEs are constantly added to the extracellular environment at a marginal rate (MGE input) simulating residual arrival from neighboring populations. The WT alleles and MGEs are degraded at a constant rate in the extracellular compartment.

Based on the model summarized in Fig. 1 and detailed in Materials and Methods, we simulated competition between several bacterial genotypes differing in their transformation rates. Population dynamics were simulated in four distinct environments: one stress-free constant environment and three environments with stochastic stress exposure, differentiated by stress frequency (see Fig. S2 in the supplemental material). Exposure to stress increases the lysis rate of susceptible cells, i.e., stresses are bactericidal, see Materials and Methods. In each tested environment, bacterial genotypes share the same eDNA pool resulting from bacterial dead cells and initially composed of wild-type (WT) alleles. A small amount of MGEs carrying a stress resistance gene were introduced to this pool. The transformable genotypes also compete with two control genotypes: a nontransformable genotype susceptible to stress (NTS) and a nontransformable resistant genotype (NTR) carrying a resistance gene that has the same cost in terms of cell replication as the MGE (see Materials and Methods).

10.1128/mBio.02443-19.2 FIG S2 Examples of stress dynamics in the three environments with stochastic stress exposure. *I* is the intensity of the stress over time for a stress frequency *F* of 5 × 10^−4^ *t*^−1^ (A), 10^−3^ *t*^−1^ (B), and 2 × 10^−3^ *t*^−1^ (C). Download FIG S2, PDF file, 0.08 MB. Copyright © 2020 Carvalho et al. 2020 Carvalho et al. This content is distributed under the terms of the Creative Commons Attribution 4.0 International license.

**FIG 2:**
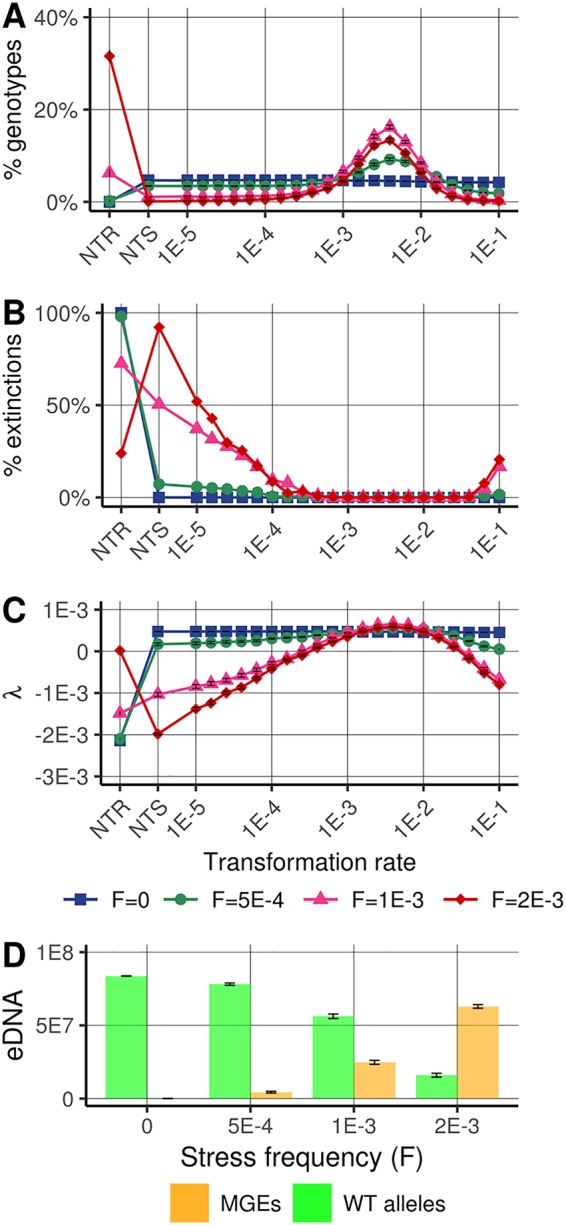
Relative success of genotypes according to their transformation strategies. (A) Proportions of the competing genotypes at *t *= 5,000: nontransformable resistant (NTR), nontransformable susceptible (NTS), and 21 genotypes with transformation rates ranging from 10^−5^ to 10^−1^ per time unit (*t*^−1^). (B) Proportions of extinction of the genotypes at *t *= 5,000, among the 500 simulations. (C) Stochastic growth rate, as proxy of the fitness of the genotypes in stochastic environments (see Materials and Methods). (D) Composition of the extracellular compartment at *t *= 5,000. Represented data are the means and standard errors calculated from 500 simulations. Population dynamics are simulated in four distinct environments: one stress-free constant environment (*F* = 0) and three environments with stochastic stress exposure, differentiated by stress frequency of *F *= 5 × 10^−4^, *F *= 10^−3^, and *F *= 2 × 10^−3^ *t*^−1^ (see Fig. S2 in the supplemental material).

**FIG 3:**
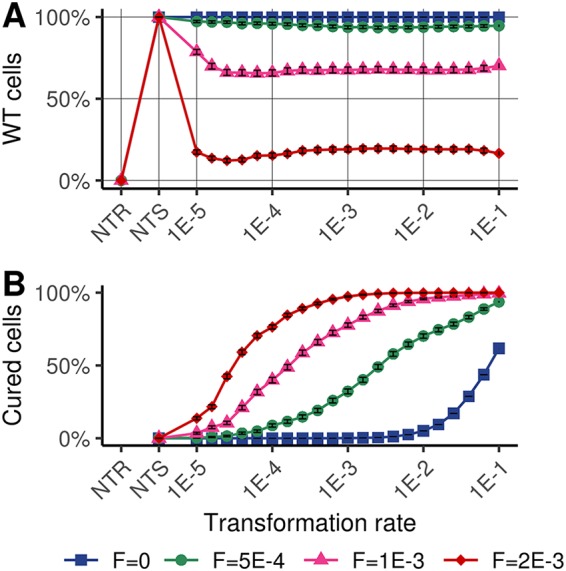
Maintenance of wild-type alleles in transformable genotypes. (A) Percentages of wild-type (WT) cells at the end of the simulations for each genotype. Extinct genotypes are not accounted for the calculus. (B) Percentages of WT cells from cure, i.e., WT cells with past MGE integration.

In the stress-free environment, all genotypes can grow when they are alone (see Fig. S3). In the competition context, the NTR genotype becomes extinct, because carrying a resistance gene reduces growth rate, while all other genotypes have similar demographic performances and do not experience extinction regardless of their transformation rate (Fig. 2A and B). This is related to the fact that there is no transformation cost in these simulations (see Fig. S4 when a cost is introduced). MGEs carrying resistance are extremely marginal in the extracellular compartment (Fig. 2D); therefore, upon transformation, cells mostly integrate WT alleles into their genome and conserve their wild-type phenotype (Fig. 3A).

10.1128/mBio.02443-19.3 FIG S3 Stochastic growth rate (λ) of the NTR, NTS, and the predominant genotype (*T*_max_ = 10^−2.4^ *t*^−1^) alone and in competition. (A) NTS performs well alone despite being susceptible to the stress but is greatly counterselected when in competition in stochastic stressful environments. (B) NTR is not affected by the stresses and performs well alone. However, it is not competitive when stresses are infrequent. (C) The genotype with an intermediate transformation rate (*T*_max_ = 10^−2.4^ *t*^−1^) performs well under all conditions. Download FIG S3, PDF file, 0.06 MB. Copyright © 2020 Carvalho et al. 2020 Carvalho et al. This content is distributed under the terms of the Creative Commons Attribution 4.0 International license.

10.1128/mBio.02443-19.4 FIG S4 Sensitivity analysis to model parameters. (A and B) Transformation cost *c*_trans_ (probability of cell lysis during a transformation event). (C and D) Type of competence trigger: constitutive competence (constant), competence induced by stress exposure (stress) or by cell density (biomass). (E and F) Mutation scenario. In blue (std), the genotypes are all in competition from the initial conditions and there is no mutation (the mutation probability *P*_mut_ = 0 and the simulation time is *t_f_* = 5 × 10^3^). In green and pink, only the NTS genotype is present at the beginning of the simulation, and the other genotypes occur randomly by mutation. For these two situations, the mutation rate is the same (*P*_mut_ = 10^−4^/replication) but the simulation time changes (*t_f_* = 2 × 10^4^ *t* and 3 × 10^4^ *t*, respectively). See Materials and Methods in main text for details. λ is the mean stochastic growth rate, and eDNA corresponds to the mean eDNA molecules at the end of simulations. Error bars are the standard errors from 200 simulations. Standard parameters (std) refer to the main text Table 1 and the stress frequency 10^−3^ *t*^−1^. Download FIG S4, PDF file, 0.3 MB. Copyright © 2020 Carvalho et al. 2020 Carvalho et al. This content is distributed under the terms of the Creative Commons Attribution 4.0 International license.

When bacterial cells are exposed to stress, even at a low frequency, the most efficient strategies that emerge are those with an intermediate transformation rate (10^−3^ < maximal transformation rate of a genotype *i* [*T*_max,_*_i_*] < 10^−2^ *t*^−1^), which have both a high abundance (no extinction) and a higher stochastic growth rate λ (i.e., geometric mean, see Materials and Methods) (Fig. 2A to C). The NTS genotype, which cannot acquire resistance genes, is disadvantaged and often goes extinct, whereas it could grow if it was alone, i.e., without competition with other genotypes (Fig. S3). The performance of genotypes with a very low transformation rate (*T*_max,_*_i_* < 10^−4^ *t*^−1^) is very similar to that of the NTS genotype even if the extinction probabilities of these genotypes are lower (Fig. 2A and B). Interestingly, and even without introducing a direct cost for transformation (in terms of replication or cell mortality), cells that transform at a very high rate (*T*_max,_*_i_* > 10^−2^ *t*^−1^) are also counterselected (Fig. 2A and B). These strategies induce very frequent changes in phenotype, including the detrimental transitions WT→resistant (MGE infected) between stresses and resistant (MGE infected)→WT during stresses (see Fig. S5).

10.1128/mBio.02443-19.5 FIG S5 Types of transformation events per cell. The graphs represent frequencies of cure (MGE infected→WT) or infections (WT→MGE infected) per cell depending on the number of stresses occurring in the simulations. Three genotypes with three transformation rates are plotted: a genotype with a very low transformation rate (*T*_max_ = 10^−5^ *t*^−1^), a genotype with an optimal transformation rate in a fluctuating environment (*T*_max_ = 10^−2.4^ *t*^−1^), and a genotype with a very high transformation rate (*T*_max_ = 10^−1^ *t*^−1^). Results presented are simulations with the standard parameters and the 4 environments presented in the main text. Download FIG S5, PDF file, 0.2 MB. Copyright © 2020 Carvalho et al. 2020 Carvalho et al. This content is distributed under the terms of the Creative Commons Attribution 4.0 International license.

Although the NTR genotype always performed well alone (Fig. S3), it was outcompeted in all environments except in a few simulations with high stress frequency (2 × 10^−3^ *t*^−1^) (Fig. 2A). This result shows that continuously carrying the resistance gene may be beneficial if the environmental stress is frequently encountered. However, despite that in the most stressful environment the NTR genotype had the highest mean total cells at the end of the simulations, it actually suffered ∼25% extinction, whereas the predominant transformable genotypes persisted in all simulations (Fig. 2B) and had a higher stochastic growth rate (λ) than NTR genotypes (Fig. 2C). The observation that the intermediate transformable genotypes outcompete the NTR genotype suggests that transformation gives a fitness advantage in stochastic environments by removing MGEs, which is costly for replication, during the periods without stress.

However, transformation is inherently risky. Transformation may lead to the acquisition of toxic and highly detrimental genes and the generation of recombination intermediates that can jeopardize chromosome integrity (39, 40). To account for such a transformation cost, we implemented a probability of cell lysis during transformation events. By including this cost (up to 10% risk of lysis during transformation), genotypes with high transformation rates were greatly impaired while strategies with intermediate transformation rates (10^−3^ < *T*_max,_*_i_* < 10^−2^ *t*^−1^) remained optimal, even if the optimum shifted toward the lower transformation rates (see Fig. S4A and B).

The competence of the cells for transformation is often regulated either by an abiotic environment (e.g., stress) or bacterial density. To explore the influence of competence regulation on the optimal transformation rate, we implemented two commonly occurring triggers of competence: stress exposure and biomass (see Materials and Methods). In the stress trigger scenario, i.e., when competence is induced by stress exposure, the optimal transformation rate increased (Fig. S4C and D). In the biomass trigger scenario, i.e., when competence is favored at high bacterial densities, genotypes with high transformation rates performed better than under a constitutive competence scenario, but the optimal genotype remained unchanged (Fig. S4C and D). To further investigate the stability of the evolutionary strategy of transformable genotypes, we tested the ability of the different genotypes (which emerge by mutation) to invade a population that initially included only the NTS genotype (see Materials and Methods). The results show that the most efficient genotypes remain those with an intermediate transformation rate, even if their invasion requires a longer simulation time (Fig. S4E and F).

In the literature, the fitness cost induced by MGEs is very variable (e.g., MGEs with antibiotic resistance genes [41]). In our simulations, when the cost of MGEs was modified, the eDNA composition of the extracellular compartment varied greatly, but the distribution of transformable genotypes remained qualitatively unchanged (see Fig. S6A and B). Moreover, the variation in the input of MGEs or in the rate of degradation of eDNA does not qualitatively change the results (Fig. S6E and F and S7A and B) or the specific accentuated degradation of extracellular MGEs (up to 10 times the one of WT alleles) (Fig. S6C and D). Finally, the optimal intermediate transformable genotypes remained stable when we increased the mean intensity or mean duration of the stresses or if the stress is bacteriostatic and not bactericidal (i.e., if stress exposure reduces the replication rate of susceptible cells instead of increasing cell lysis; see Materials and Methods) (see Fig. S8). Overall, the results point out that intermediate transformation rates are extremely efficient strategies for buffering environmental stochasticity in many ecological contexts.

10.1128/mBio.02443-19.6 FIG S6 Sensitivity analysis to model parameters. (A and B) Fitness costs induced by MGE *c*_MGE_ (growth reduction of the host). (C and D) Decay rates of extracellular MGE *R*_MGE_, creating an asymmetry with the degradation of extracellular WT alleles, which remains at the standard value 0.15. (E and F) Residual inputs of MGE molecules in the extracellular compartment *M*_input,MGE_. See Materials and Methods in main text for details. λ is the mean stochastic growth rate, and eDNA corresponds to the mean eDNA molecules at the end of simulations. Error bars are the standard errors from 200 simulations. Standard parameters (std) refer to the main text Table 1 and the stress frequency 10^−3^ *t*^−1^. Download FIG S6, PDF file, 0.3 MB. Copyright © 2020 Carvalho et al. 2020 Carvalho et al. This content is distributed under the terms of the Creative Commons Attribution 4.0 International license.

10.1128/mBio.02443-19.7 FIG S7 Sensitivity analysis to model parameters. (A and B) eDNA decay rate *R*_DNA_ of both extracellular WT alleles and MGEs. (C and D) Carrying capacities of the environment *K.* The size of the initial cell population is *K*/10. (E and F) Simulation time *t_f_*. See Materials and Methods in main text for details. λ is the mean stochastic growth rate, and eDNA corresponds to the mean eDNA molecules at the end of simulations. Error bars are the standard errors from 200 simulations. Standard parameters (std) refer to the main text Table 1 and the stress frequency 10^−3^ *t*^−1^. Download FIG S7, PDF file, 0.3 MB. Copyright © 2020 Carvalho et al. 2020 Carvalho et al. This content is distributed under the terms of the Creative Commons Attribution 4.0 International license.

10.1128/mBio.02443-19.8 FIG S8 Sensitivity analysis to model parameters. (A and B) Mean stress duration *d*_mean_. (C and D) Mean stress intensity *I*_mean_. (E and F) Growth inhibition during stress *G_inh_*. When *G_inh_* is >0, the stress is considered bacteriostatic and reduces growth by the factor (1 − *G_inh_*) but does not affect lysis rate. See Materials and Methods in main text for details. λ is the mean stochastic growth rate, and eDNA corresponds to the mean eDNA molecules at the end of simulations. Error bars are the standard errors from 200 simulations. Standard parameters (std) refer to the main text Table 1 and the stress frequency 10^−3^ *t*^−1^. Download FIG S8, PDF file, 0.3 MB. Copyright © 2020 Carvalho et al. 2020 Carvalho et al. This content is distributed under the terms of the Creative Commons Attribution 4.0 International license.

### The competitiveness of transformable cells relies on the reversible integration of MGEs.

To examine the importance of chromosomal curing in the success of genotypes with intermediate transformation rates (Fig. 2A to C) (transformation rates 10^−3^ < *T*_max,_*_i_* < 10^−2^ *t*^−1^), we determined the phenotypic composition (proportion of WT cells and cells infected by MGEs) for the different genotypes (Fig. 3A) as well as the proportion of WT cells from parent cells that previously had an MGE in their genome (WT cells from cure) (Fig. 3B). The proportions of WT cells were similar in all transformable genotypes despite genotypes being represented in various proportions (Fig. 2A). The proportion of WT cells, however, decreased as the stress frequency increased. By determining the origin of WT cells in stochastic stressful environments, we found that most of them originated from genome cure for the predominant genotypes (Fig. 3B) (transformation rates between 10^−3^ and 10^−2^ *t*^−1^). The proportion of WT cells from cure increases with the frequency of stress exposure (Fig. 3B).

**FIG 4:**
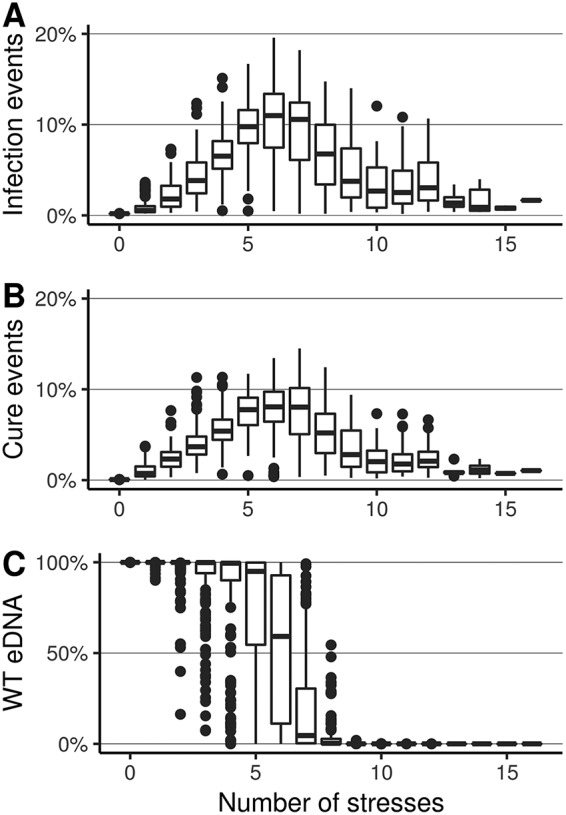
Types of transformation events (infection or cure) for the predominant genotype (with transformation rate 10^−2.4^ *t*^−1^). (A) Box plot of the percentages of cure events (MGE infected cell→WT cell) among all transformation events depending on the number of stresses. (B) Box plot of the percentage events of infection by MGE (WT cell→MGE infected cell) among all transformation events depending on the number of stresses. Results group simulations with all stress frequencies. (C) Composition of the extracellular compartment at *t *= 5,000 (box plot of the percentage of WT alleles).

These results show that transformation, performed at an intermediate rate, is a powerful mechanism for regenerating the WT genotype in stochastic environments. From the analysis of transformation events performed by the dominant genotype (*T*_max,_*_i_* = 10^−2.4^ *t*^−1^), we find that the switch of phenotypes (WT→resistant or resistant→WT) occurs mainly when the genotype faces intermediate numbers of stresses during simulations (∼4 to 7 stresses for 5,000 time units) (Fig. 4A and B), which also corresponds to a greater eDNA diversity in the extracellular compartment (Fig. 4C). With extreme number of stresses, the environment is either very little disturbed or, conversely, frequently disturbed, and the great majority of transformation events are neutral since they replace the DNA of the cells with another identical DNA (Fig. 4).

## DISCUSSION

While transformation is often considered a mechanism sustaining bacterial genetic diversification and mixing, recent investigations suggest that its primary function would be to cure bacterial genomes of their infectious parasitic genetic elements (18, 35). We show that when bacteria face a fluctuating environment containing temporarily beneficial MGEs (costly MGEs for bacterial growth but carrying stress resistance genes), these two points of view can be unified in a common framework. In our simulations, genotypes with an intermediate transformation rate, when competing with other genotypes, have a large selective advantage in fluctuating environments (stochastic stress exposure) by maximizing the probability of genotype persistence and their stochastic growth rate (Fig. 2B and C). In our simulations, these intermediate transformation rates favor the random acquisition of extracellular MGEs carrying resistance genes, which allows genotypes to continue to grow during periods of environmental stress. Furthermore, these same transformation strategies allow MGE removal (genome cure) after each stress episode and thus the reconstitution of the initial genome, which is beneficial because maintaining MGEs is costly in terms of replication for host cells (42). Overall, our results suggest that intermediate transformation rates, by generating reversible integration of MGEs, stabilize over the long term the genotypes and genomes of bacteria that evolve in a stochastic environment.

Bacterial populations often face variable environments and are exposed to a wide variety of unpredictable stresses (e.g., heavy metals and antibiotics). The most widely proposed adaptation mechanisms to deal with them correspond to the diversified bet hedging—stochastic switching between phenotypic states (43–46)—corresponding to a risk-spreading strategy that facilitates genotype invasion and persistence in the face of unpredictable fluctuating environmental conditions (47). The most common example corresponds to the production of both reproductive individuals (replicative cells in bacteria) and individuals that remain in a dormant state for a more or less prolonged period of time (or “persister” cells in bacteria) (48, 49). Similarly, transformation can be seen as a risk-spreading strategy by randomly producing new phenotypes from MGE integration, then reconstituting the initial genotype by MGE removal. Interestingly, this strategy would enable coping with a wide variety of stresses (by successively integrating and removing different MGEs) while maintaining active (replicative) cells during and between periods of stress. In this sense, transformation could be one of the most efficient risk-spreading strategies in a stochastic environment, which could explain its ubiquity in the bacterial phylum (see Fig. S1 in the supplemental material).

We aimed to simulate transformation rates with orders of magnitude coherent with empirical observations (50). However, empirical estimation of the transformation rates in the laboratory is scarce—and almost impossible in the field—because (i) the state of competence in bacteria is often dependent on environmental conditions with differing triggering factors between strains (37, 51), (ii) the estimation of the transformation rate does not account for transformation events replacing DNA with an identical allele, which is the majority of the transformation events in our simulations (Fig. 4), and (iii) the transformation rate depends on the characteristics of the imported DNA, such as its size but also the capacity of few MGEs to inhibit transformation once acquired (51). Because of these limitations, in our study, we considered a wide range of transformation rates, and we show that, when in competition, genotypes with transformation rates too low or too high should be counterselected because they generate too little or too much phenotypic change, often leading to the formation of phenotypes that are not adequate for environmental conditions (cells with MGE in stress-free period or WT cells during exposure to stress) (Fig. S5). Thus, the wording “intermediate transformation rate” used in the manuscript is likely to be applicable to natural populations, but an extensive meta-analysis or experimental screening of natural isolates would be necessary to know the distribution of transformation rates in natural environments and to confirm this inference. However, it is already known that mutants displaying phenotypes of elevated transformability are easily isolated in transformable species under laboratory conditions (52–56). Such a hypertransformable phenotype is due to mutations causing upregulation of the transformation system or alteration of components of the competence machinery. Interestingly, these hypertransformable phenotypes are not common in natural isolates, suggesting that they can arise but are quickly counterselected. Our prediction is also consistent with the fact that in many transformable species, only a fraction of cells of the same genotype are in the state of competence and are likely to transform at the same time, even if all cells are under controlled laboratory conditions favorable for transformation (8, 57–59). This heterogeneity in competence states, which would correspond to the intermediate transformation rates in our model, should greatly contribute to the spreading of risks in a stochastic environment.

While our model aims to be generalist, it is difficult to determine the extent to which the optimal transformation rate found is specific to strain-environment-MGE combinations. However, the sensitivity analysis carried out shows that the optimal intermediate transformation rate is robust in the large range of parameters tested (Fig. S6 to 8), including when rare transformable mutants have to invade a nontransformable population (Fig. S4E and F). When competence is triggered by high cell density, the optimal transformation rate remains unchanged even if genotypes with a high transformation rate have a higher fitness than when the competence is constitutively expressed (Fig. S4C and D). When competence is triggered by stress exposure, the optimal transformation rate increases. In this latter situation, cells are rarely in a state of competence (exposure to stress being rare), and when cells are exposed to stress, high transformation rates would confer a selective advantage by increasing the chances of capturing MGEs carrying resistance genes (which are relatively rare in the extracellular environment at the beginning of stress exposure).

According to our proposal, the extracellular compartment would constitute a reservoir of MGEs, providing bacteria with a “communal gene pool” (60), which should be highly fluctuating in its composition. In our model, we introduced MGEs at an extremely low rate, simulating the residual intake of MGEs from other nearby bacterial populations. The proportion of these MGEs remains extremely low when stress is rare or absent (Fig. 2D), while they can become extremely abundant in the extracellular compartment when stress exposure becomes frequent (Fig. 2D). Moreover, the spatial distribution of eDNA could be heterogeneous, and transformation rates may themselves fluctuate spatially within the same population (e.g., in or out of biofilms [61]). Cells close to the spatial boundaries of an isogenic population could tend to acquire more foreign DNA and cells in the center more kin DNA, making subpopulations more subject to MGE acquisition or removal and subject to variable transformation triggers. Our work then encourages empirical analysis of the spatiotemporal dynamics of the extracellular compartment whose composition should depend on the regime of environmental fluctuations (intensity, duration, and frequency of exposure to stress) but also on the connectivity between bacterial populations and the degree of persistence of MGEs or wild alleles according to their characteristic (e.g., their size). With the advent of methods to study bacteria-MGE interactions in complex microbiota (62, 63), contrasting antibiotic treatments could be an ideal experimental design to explore the dynamics of this extracellular reservoir and its consequences on the spread of resistance genes. For example, permanent antibiotic treatment could enrich the extracellular compartment with MGEs to such an extent that bacteria can no longer cure their genome through transformation. This point of view sheds new light on our understanding of (and fight against) the spread of antibiotic resistance in hospitals and potentially paves the way to new strategies for fighting antimicrobial resistance.

Independently of the transient acquisition of MGEs carrying resistance genes, there are undoubtedly empirical facts in favor of the perennial acquisition of new genes from different species by natural transformation (64, 65). We propose that such perennial integration should be the result of rare and “accidental” transformation events leading to the formation of new bacterial strains potentially in competition with the parent strain. This process should involve coevolution mechanisms such as compensatory mutations to improve the stability of the MGE-host couple long term (66). Therefore, one of the main evolutionary causes of transformation should be to generate reversible integration of MGE to buffer environmental stochasticity, while the perennial acquisition of new genes should be a by-product of transformation (or an exaptation) only exceptionally occurring, when bacteria face new but lasting environmental conditions. These accidental transfers would, however, play a key role in the diversification of bacterial lines and would be of the same order as horizontal transfers observed in eukaryotes, which are rare but have a profound impact on their evolution (67, 68).

Contrary to our proposal, the “chromosomal curing” hypothesis focuses on the removal of infectious MGEs, which parasitize bacterial genomes (18). This point of view is particularly relevant in the case of high selective pressure driven by infectious phages. However, the chromosomal curing hypothesis alone cannot explain the frequent observations of accumulation of resistance genes in MGEs transferable by transformation, such as resistance islands which can be very numerous and diversified (42, 69). Here, we propose that bacteria, through transformation, actively exploit specific categories of MGEs, such as transposons, integrons, gene cassettes, and genomic islands, that can be very variable in their composition in resistance genes and can be integrated transiently into the bacterial genome. However, further investigations will be needed to study the interactions between the different evolutionary functions of transformation by considering both fluctuating stress exposure and various classes of MGEs, such as infectious phages, integrative MGEs carrying stress resistance genes, or conjugative elements.

In conclusion, in this work, we point out that transformation, which is a widespread trait, allows the transient acquisition of MGEs carrying stress resistance genes, which increases bacterial fitness under stochastic stress exposure. Because many bacterial species are probably frequently exposed to such fluctuating environments, this function could be often operational. However, it must be evaluated both empirically and theoretically in different ecological contexts and in interaction with the other functions already proposed. Here, our work is focused on natural transformation, which is the most common mechanism of HGT capable of generating chromosomal gene replacement. Yet, our conclusion may also apply to any other mechanism which promotes large chromosomal recombination events between individuals of bacterial populations. Namely, it may also explain the presence of the “distributive conjugal transfer” in one of the rare family of bacteria, the *Mycobacteriaceae*, which lack *ComEC* and the transformation system (70). Understanding the evolution of bacterial populations and communities in a fluctuating environment will also require addressing the coevolution of bacteria and MGEs by considering a possible alternation of genetic conflicts and cooperation between them. This perspective could help understand and prevent the spread of antibiotic resistance in bacterial populations and communities.

## MATERIALS AND METHODS

We developed a stochastic computational model which includes two types of compartments: bacterial cells and extracellular DNA (eDNA), similarly to previous models (18). The overall structure of the model is displayed in Fig. 1. Bacterial cells have an insertion site in their chromosome which can be occupied by two DNA types: a wild type (WT) allele and a costly MGE conferring stress resistance. In the population, 23 genotypes (*i*) compete with each other, among which 21 genotypes (*i*) differ in their maximal transformation rates (*T*_max,_*_i_*). We also introduced two control nontransforming genotypes, one with the WT allele and one with the stress resistance allele, named NTS and NTR, respectively. The NTR genotype is initialized with cells carrying the stress resistance allele and its associated cost. The transformable and the NTS genotypes are initialized with WT cells only, i.e., cells carrying the WT allele. Genotypes, according to their transformation strategy, are equally distributed at the beginning of the simulations, with an initial population size *N*_0_ = *K*/10 cells, with *K* the carrying capacity. Bacterial growth follows a logistic growth model, with a carrying capacity *K *= 10^7^ cells (i.e., the maximum number of cells that the habitat can support). The number of replicating cells per genotype *i* with allele *j* and per time step *dt*, *G_i_*_,_*_j_*_,_*_t_*_+1_, is determined using a binomial distribution:

(1) $$G_{i,j,t+1}∼\mathrm{Bin}\left(\mu_{j,t}\mathrm{.dt},N_{i,j,t}\right)$$

(2) $${\text{μ}}_{j,t}=\left({\text{μ}}_{\text{max}}-\frac{{\text{μ}}_{\text{max}}-k_{b}}{K}N_{\text{tot,}t}\right)*\left(1-c_{j}\right)$$

*N_i_*_,_*_j_*_,_*_t_* is the number of cells with the genotype *i* carrying the DNA type *j* at time *t.* μ*_j_*_,_*_t_* is the replication rate of cells with the DNA type *j* at time *t.* μ_max_ is the maximal growth rate. *k_b_* is the constant basal lysis rate, independent of the presence of stress. *N*_tot,_*_t_* is the total number of cells in the population (considering all genotypes) at time *t*, and *c_j_* is the cost induced by the DNA type *j* on the replication of cells (*c*_WT_ = 0 and *c*_MGE_ > 0). The number of lysed cells per time step *L_i_*_,_*_j_*_,_*_t_*_+1_ is calculated using a binomial distribution:

(3) $$L_{i,j,t+1}∼\mathrm{Bin}\left(k_{j,t}\mathrm{.dt},N_{i,j,t}\right)$$

(4) $$k_{j,t}=k_{b}+I_{S,t}*\left(1-r_{j}\right)$$

*k_j_*_,_*_t_* is the lysis rate of cells with the DNA type *j* at time *t*. *I_S_*_,_*_t_* is the intensity of the stress at time *t*. *r_j_* is the stress resistance provided by the allele *j*. Stresses increase the lysis rate of cells carrying a wild-type allele, whereas the lysis rate of cells carrying an MGE remains at the basal rate *k_b_* (*r*_WT_ = 0 and *r*_MGE_ = 1). The number of competent cells undergoing a transformation event during a time step *C_i_*_,_*_j_*_,_*_t_*_+1_ is determined using a binomial distribution:

(5) $$C_{i,j,t+1}∼\mathrm{Bin}\left(T_{i,t}\mathrm{.dt},N_{i,j,t}\right)$$

(6) $$T_{i,t}=T_{\text{max},i}*\left(\frac{\text{α}A_{\text{tot},t}}{1+\text{α}A_{\text{tot},t}}\right)$$

*T_i_*_,_*_t_* is the transformation rate at time *t*. *T*_max,_*_i_* is the maximal transformation rate of the genotype *i* and is the only parameter differentiating the 21 transformable genotypes. α is the binding rate between cells and eDNA. *A*_tot,_*_t_* is the total number of eDNA at time *t*. The probability of a cell to take up a particular type of eDNA is proportional to the DNA composition of the extracellular compartment. Cells undergoing a transformation event change their genotype accordingly to the DNA type integrated. The overall variation of a genotype *i* containing a DNA type *j* (WT or MGE) during a time step is summarized by:

(7) $$N_{i,j,t+1}=N_{i,j,t}+G_{i,j,t+1}-L_{i,j,t+1}-C_{i,j,t+1}^{j\to !j}+C_{i,!j,t+1}^{!j\to j}$$

In the extracellular compartment, eDNA is degraded at a constant rate *R_j_*. The number of degraded eDNA molecules per time step *D_j_*_,_*_t_*_+1_ is determined using a binomial distribution:

(8) $$D_{j,t+1}∼\mathrm{Bin}\left(R_{j}\mathrm{.dt},A_{j,t}\right)$$

*A_j_*_,_*_t_* is the number of eDNAs of type *j*. The extracellular compartment is alimented by eDNA from lysed cells, each lysed cell releasing a DNA molecule corresponding to its DNA. In addition, eDNA is added at a constant rate to the extracellular compartment (open system). The number of eDNA molecules *j* added per time step *M_j_* is defined by:

(9) $$M_{j}=M_{\text{input,}j}*\mathrm{dt}$$

*M*_input,_*_j_* is the number of molecules of eDNA of type *j* added per time unit. Only MGEs are added this way in the extracellular compartment (*M*_input,WT_ = 0). *M*_input,MGE_ is set to be residual, that is, orders of magnitude lower than the DNA released by cell lysis. The overall variation of a DNA of type *j* in the extracellular compartment during a time step is summarized by:

(10) $$A_{j,t+1}=A_{j,t}-D_{j,t+1}+M_{\text{input},j}+\overset{n}{\underset{i=1}{\sum }}\left[L_{i,j,t+1}+C_{i,j,t+1}^{j\to !j}-C_{i,!j,t+1}^{!j\to j}\right]$$

Bacteria are exposed to random stresses affecting the lysis rate of WT cells and occurring with a frequency *F*_stress_. The probability of a stress to start during a time step is *F*_stress_ *× dt*. When a stress starts, another one cannot begin before the first one ended. The duration *d_S_* and intensity *I_S_* of each stress are randomly drawn from a normal distribution with the means and standard deviations *d*_mean_, *d*_SD_, *I*_mean_, and *I*_SD_. To estimate the efficiency of a genotype in fluctuating environments, we calculate its stochastic growth rate λ as the logarithm of the geometric mean:

(11) $$\lambda =\frac{1}{t_{f}}*\ln\left(\frac{N_{f,i}+1}{N_{0,i}}\right)$$

with *t_f_* as the simulation time, *N_f_*_,_*_i_* as the final number of cells of the genotype *i*, and *N*_0,_*_i_* as the initial number of cells of the genotype *i*.

The summary of the parameters used is presented in Table 1. The order of magnitude of the parameters corresponds to that from previous computational models of transformation (18, 24). The transformation rates of the genotypes in our simulated bacterial community ranged from 10^−5^ *t*^−1^ to 10^−1^ *t*^−1^, which is in the range of previous models. With the basal lysis rate *k_b_* = 0.2 *t*^−1^ and the simulation time *t_f_* = 5,000*t*, approximately 1,000 cell generations are produced per simulation in antibiotic-free environments. The system does not reach equilibrium with *t_f_* = 5,000*t*, but increasing simulation time only mildly influences simulation outcomes (see Fig. S7E and F in the supplemental material). The carrying capacity *K* is set to 10^7^ cells, but diminishing *K* could increase the selection pressure on genotypes and increasing *K* could reduce selection pressure, i.e., the distribution of genotypes could be more narrow or wide but remains qualitatively similar (Fig. S7C and D). The mean stress intensity used (*I*_mean_ = 0.5 *t*^−1^) significantly increases the death rate above the maximal growth rate (*k_b_ + I*_mean_ ≫ μ_max_), characteristic of lethal stresses such as bactericidal antibiotic concentrations. In addition, we considered a scenario where the antibiotic is bacteriostatic (Fig. S8E and F). In this case, the lysis rate *k_j_*_,_*_t_* remains constant (*k_j_*_,_*_t_* = *k_b_*), but the growth rate of susceptible cells μ_WT,_*_t_* is reduced by a factor (1 − *G_inh_*) during stress exposure.

**TABLE 1:** Default parameters used

Symbol	Default value	Unit	Description
*N*_0_	10^6^	Cells	Initial number of wild type cells (split between genotypes)
μ_max_	0.3	*t*^−1^	Maximal growth rate
*k_b_*	0.2	*t*^−1^	Basal lysis rate
*K*	10^7^	Cells	Carrying capacity of the environment
*c*_WT_	0	%	Fitness cost of WT alleles
*c*_MGE_	5	%	Fitness cost of MGEs (growth rate reduction)
*r*_WT_	0	%	Resistance carried by WT alleles
*r*_MGE_	100	%	Resistance carried by MGEs
*T*_max,_*_i_*	Specified	*t*^−1^	Maximal transformation rate of a genotype i
α	4 × 10^−5^	*t*^−1^	Binding rate cell/eDNA
*c*_trans_	0	NA^*a*^	Transformation cost (lysis probability per transformation events
*F*_stress_	Specified	*t*^−1^	Stress frequency
*I*_mean_	0.5	*t*^−1^	Mean stress intensity (death rate increase)
*I*_SD_	0.05	*t*^−1^	Standard deviation stress intensity
*d*_mean_	100	*t*	Mean stress duration
*d*_SD_	10	*t*	Standard deviation stress duration
*R*_WT_	0.15	*t*^−1^	Decay rate of the extracellular wild type alleles
*R*_MGE_	0.15	*t*^−1^	Decay rate of the extracellular MGEs
*M*_input,WT_	0	Molecule·*t*^−1^	Input of WT alleles in the extracellular compartment
*M*_input,MGE_	10^3^	Molecule·*t*^−1^	Input of MGEs in the extracellular compartment
*P*_mut_	0	Replication^−1^	Probability to switch genotype during cell replication
*t_f_*	5,000	*t*	Duration of one simulation
*dt*	0.01	*t*	Time step

a NA, not applicable.

We also considered two scenarios with regulated transformation, transformation triggered by stress or by biomass (Fig. S4C and D). In the stress trigger scenario, the transformation rate *T_i_*_,_*_t_* is weighted by the factor *I_S_*_,_*_t_/I*_mean_, i.e., transformation is activated during stresses:

(12) $$T_{i,t}=T_{\text{max,}i}*\left(\frac{\text{α}A_{\text{tot},t}}{1+\text{α}A_{\text{tot,}t}}\right)*\frac{I_{S,t}}{I_{\text{mean}}}$$

In the biomass trigger scenario, the transformation rate *T_i_*_,_*_t_* is weighted by the factor *N*_tot,_*_t_/K*, i.e., transformation is reduced during stresses if the cell population collapses:

(13) $$T_{i,t}=T_{\text{max,}i}*\left(\frac{\text{α}A_{\text{tot,}t}}{1+\text{α}A_{\text{tot,}t}}\right)*\frac{N_{\text{tot},t}}{K}$$

Finally, we considered an evolutionary scenario to test the invasiveness of the different genotypes which emerge by mutation in a population initially composed exclusively of the NTS genotype (Fig. S4E and F). With this “mutation scenario,” simulations are initialized with only NTS cells, and each cell has a probability *P*_mut_ at each replication to randomly switch to one of the 22 other genotypes. The number of genotype switches *S_i_*_,_*_j_*_,_*_t_*_+1_ depends on the number of replicating cells in the same time step *G_i_*_,_*_j_*_,_*_t_*_+1_ and the mutation probability *P*_mut_:

(14) $$S_{i,j,t+1}∼\mathrm{Bin}\left(P_{\text{mut}},G_{i,j,t+1}\right)$$

Muted cells are moved from the genotype *i* to another, which modifies equation 7 accordingly. Genotypes conserve their DNA type *j* upon switching; consequently, the NTR and NTS genotypes cannot switch between each other but can emerge from transformable genotypes.
