# Supplementary material for: Bacterial Transformation Buffers Environmental Fluctuations through the Reversible Integration of Mobile Genetic Elements
Source: mBio. 2020 Mar 3;11(2):e02443-19. doi: 10.1128/mBio.02443-19 (PMC7064763; doi:10.1128/mBio.02443-19)
Supplement: FIG S1 [file mBio.02443-19-sf001.pdf]

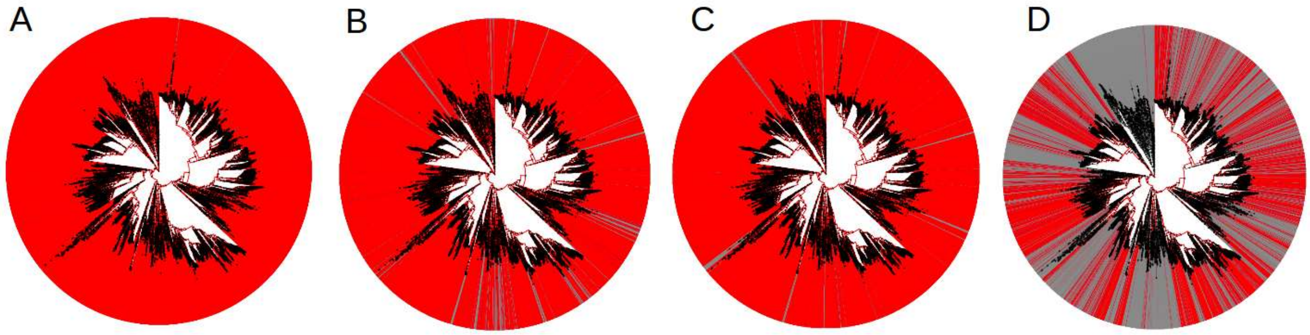

Sup.Figure 1: Proportion of competence genes in the AnnoTree database. Graphs represent the phylogenic tree of the 23,936 bacterial genomes. Red lines indicate the gene has been found in the genome. (A) *recA* (KEGG identifier K03553), 23059 genome hits; *recA* is a gene involved in homologous recombination, a core function essential to chromosome maintenance, and is well known for its ubiquity in all bacterial genomes. (B) and (C) are genes required for transformation, specifically expressed during competence, and are therefore indicators of the ability to perform natural transformation: *comEC* (KEGG identifier K02238), 20171 genome hits, involved in the uptake of extracellular DNA and (C) *drpA* (KEGG identifier K04096), 21523 genome hits, involved in protection of the incoming DNA and its integration in the recipient genome. DprA acts by directly and specifically interacting with ssDNA imported through the ComEC channel and then brings to RecA for recombination. Involvement of DprA in processing of ssDNA other than transforming DNA have so far been excluded (generic recombination). (D) In contrast, the distribution of *fliC*, (KEGG identifier K02406), 9299 genome hits, encoding the subunit of the bacterial flagellum, is consistent with an accessory function.
