# Supplementary material for: Bacterial Transformation Buffers Environmental Fluctuations through the Reversible Integration of Mobile Genetic Elements
Source: mBio. 2020 Mar 3;11(2):e02443-19. doi: 10.1128/mBio.02443-19 (PMC7064763; doi:10.1128/mBio.02443-19)
Supplement: FIG S2 [file mBio.02443-19-sf002.pdf]

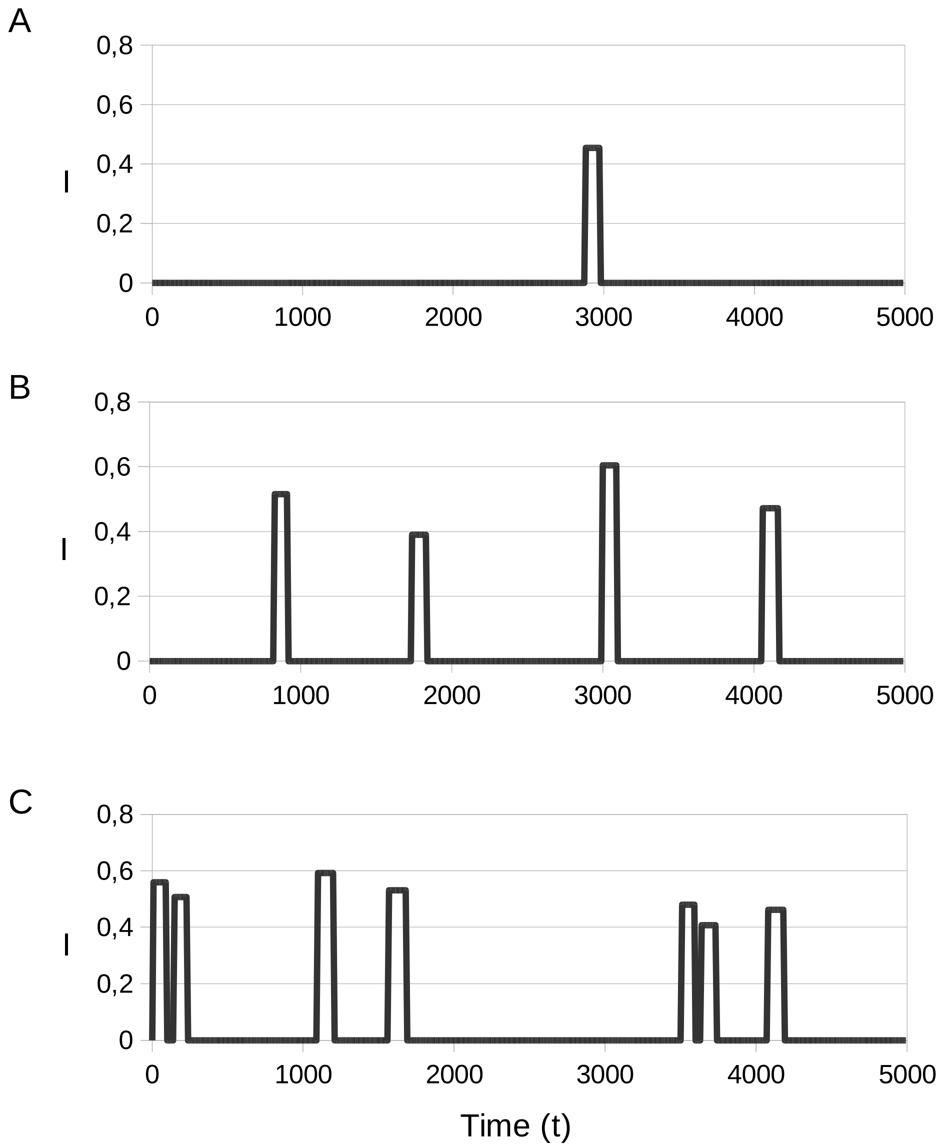

Sup. Figure 2: Examples of stress dynamics in the three environments with stochastic stress exposure. *I* is the intensity of the stress over time for a stress frequency *F* of (A) $5*10^{-4}$ t^{-1} (B) 10^{-3} t^{-1} and (C) $2*10^{-3}$ t^{-1}.
