# Supplementary material for: Bacterial Transformation Buffers Environmental Fluctuations through the Reversible Integration of Mobile Genetic Elements
Source: mBio. 2020 Mar 3;11(2):e02443-19. doi: 10.1128/mBio.02443-19 (PMC7064763; doi:10.1128/mBio.02443-19)
Supplement: FIG S3 [file mBio.02443-19-sf003.pdf]

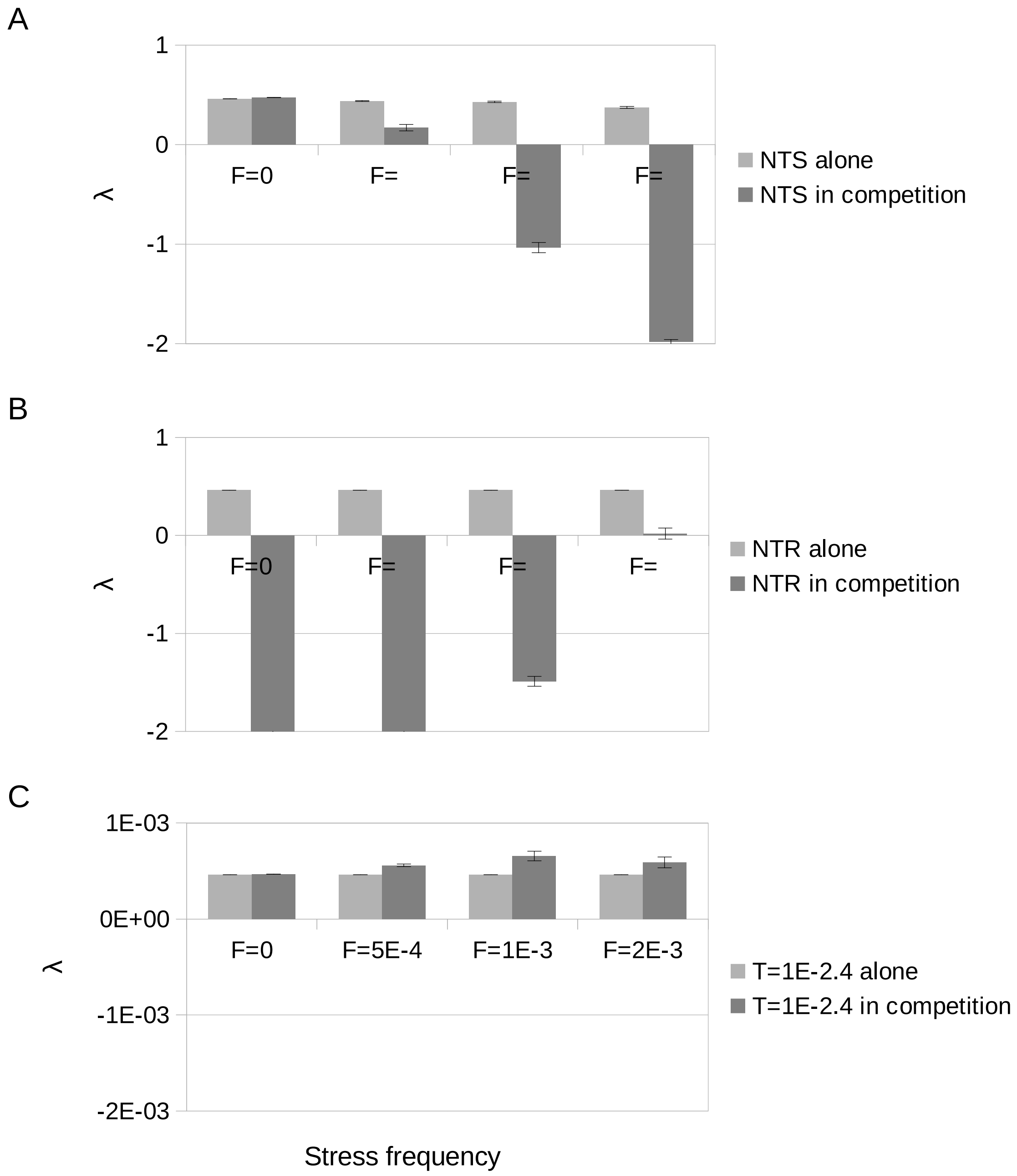

Sup. Figure 3: Stochastic growth rate (λ) of the NTR, NTS and the predominant genotype ($T_{max}=10^{-2.4}t^{-1}$) alone and in competition. (A) NTS performs well alone despite being susceptible to the stress but is greatly counter selected when in competition in stochastic stressful environments. (B) NTR is not affected by the stresses and performs well alone. However, it is not competitive when stresses are infrequent. (C) The genotype with an intermediate transformation rate ($T_{max}=10^{-2.4}t^{-1}$) performs well in all conditions.
