# Supplementary material for: Bacterial Transformation Buffers Environmental Fluctuations through the Reversible Integration of Mobile Genetic Elements
Source: mBio. 2020 Mar 3;11(2):e02443-19. doi: 10.1128/mBio.02443-19 (PMC7064763; doi:10.1128/mBio.02443-19)
Supplement: FIG S4 [file mBio.02443-19-sf004.pdf]

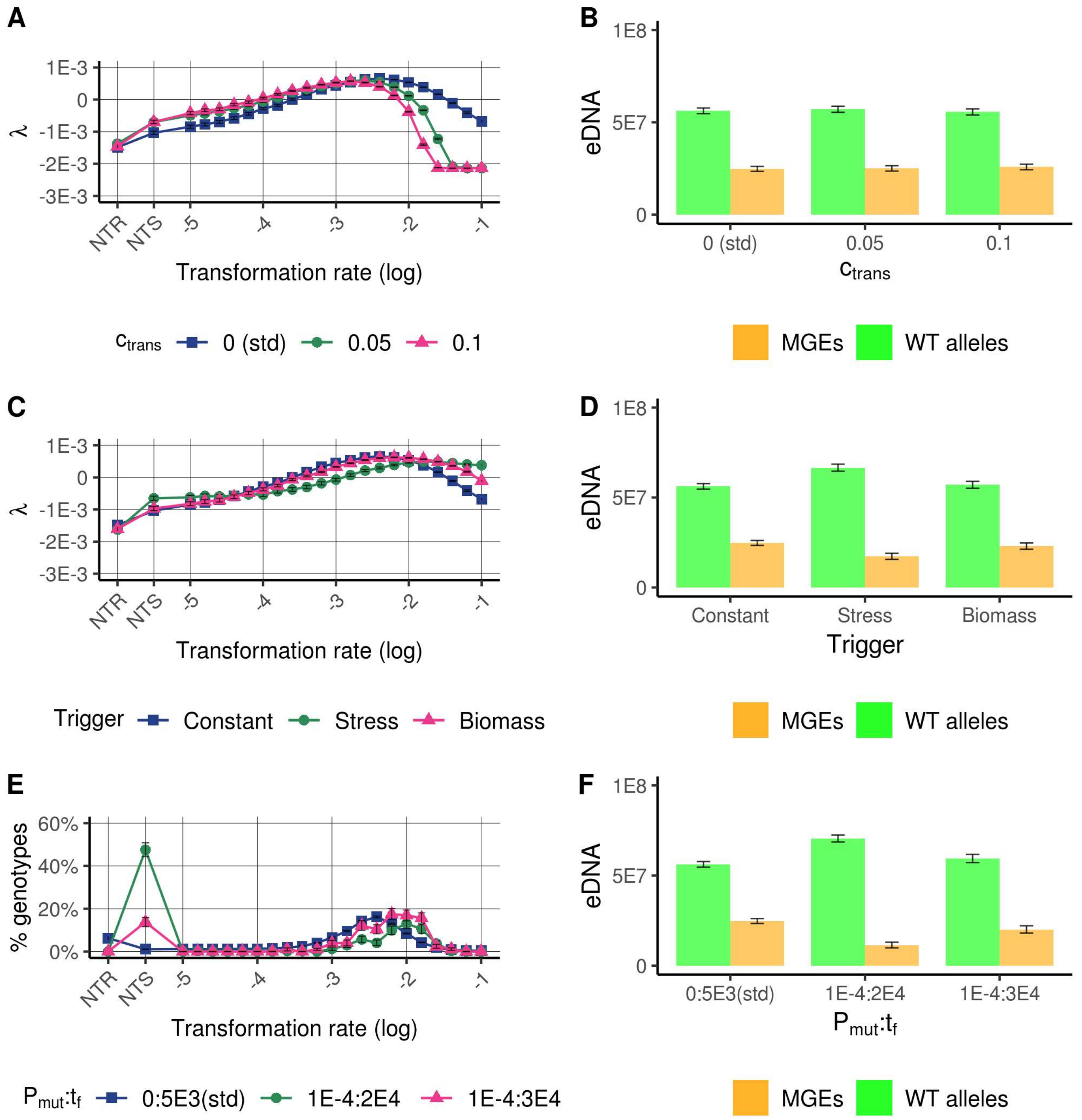

Sup.Figure 4: Sensitivity analysis to model parameters. (A,B) Transformation cost c_{trans} (probability of cell lysis during a transformation event). (C,D) Type of competence trigger: constitutive competence (Constant), competence induced by stress exposure (Stress) or by cell density (Biomass). (E,F) Mutation scenario. In blue (std), the genotypes are all in competition from the initial conditions and there is no mutation (the mutation probability P_{mut}=0 and the simulation time is $t_f=5*10^3$). In green and pink, only the NTS genotype is present at the beginning of the simulation and the other genotypes occur randomly by mutation. For these two situations the mutation rate is the same ($P_{mut}=10^{-4}$/replication) but the simulation time changes ($t_f=2*10^4$t and $3*10^4$t respectively). See Methods in main text for details. λ is the mean stochastic growth rate and eDNA correspond to the mean eDNA molecules at the end of simulations. Error bars are the standard error of 200 simulations. Standard parameters (std) refer to the main text Table 1 and the stress frequency $10^{-3}t^{-1}$.
