# Supplementary material for: Bacterial Transformation Buffers Environmental Fluctuations through the Reversible Integration of Mobile Genetic Elements
Source: mBio. 2020 Mar 3;11(2):e02443-19. doi: 10.1128/mBio.02443-19 (PMC7064763; doi:10.1128/mBio.02443-19)
Supplement: FIG S5 [file mBio.02443-19-sf005.pdf]

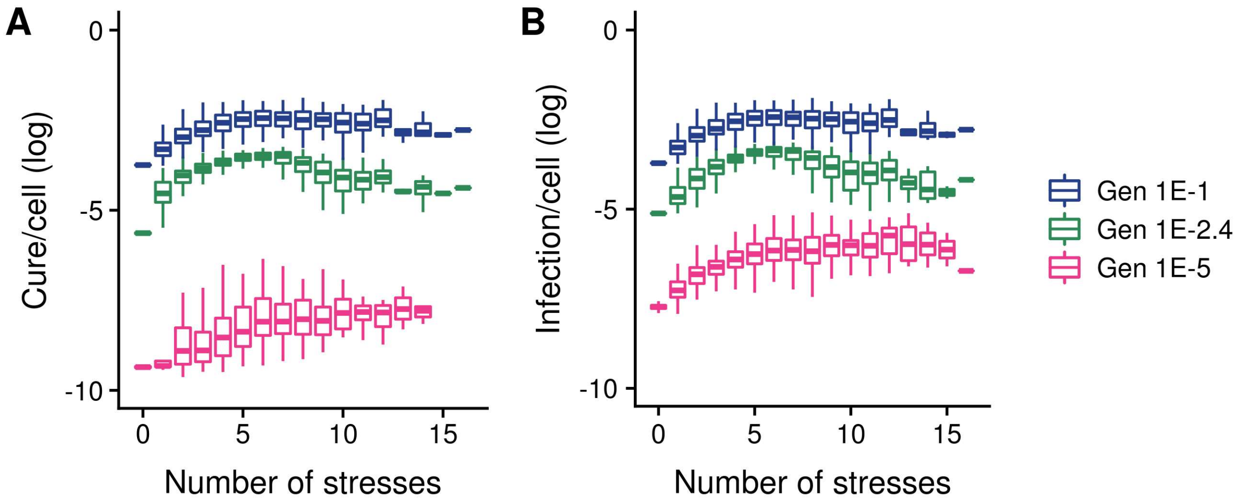

Sup. Figure 5: Types of transformation events per cell. The graphs represent frequency of cure (MGE infected ->WT) or infections (WT→MGE infected) per cell depending on the number of stresses occurring in the simulations. Three genotypes with three transformation rates are plotted: a genotype with a very low transformation rate ($T_{max}=10^{-5}\,t^{-1}$), a genotype with an optimal transformation rate in a fluctuating environment ($T_{max}=10^{-2.4}\,t^{-1}$) and a genotype with a very high transformation rate ($T_{max}=10^{-1}\,t^{-1}$). Results presented are simulations with the standard parameters and the 4 environments presented in the main text.
