# Supplementary material for: Bacterial Transformation Buffers Environmental Fluctuations through the Reversible Integration of Mobile Genetic Elements
Source: mBio. 2020 Mar 3;11(2):e02443-19. doi: 10.1128/mBio.02443-19 (PMC7064763; doi:10.1128/mBio.02443-19)
Supplement: FIG S6 [file mBio.02443-19-sf006.pdf]

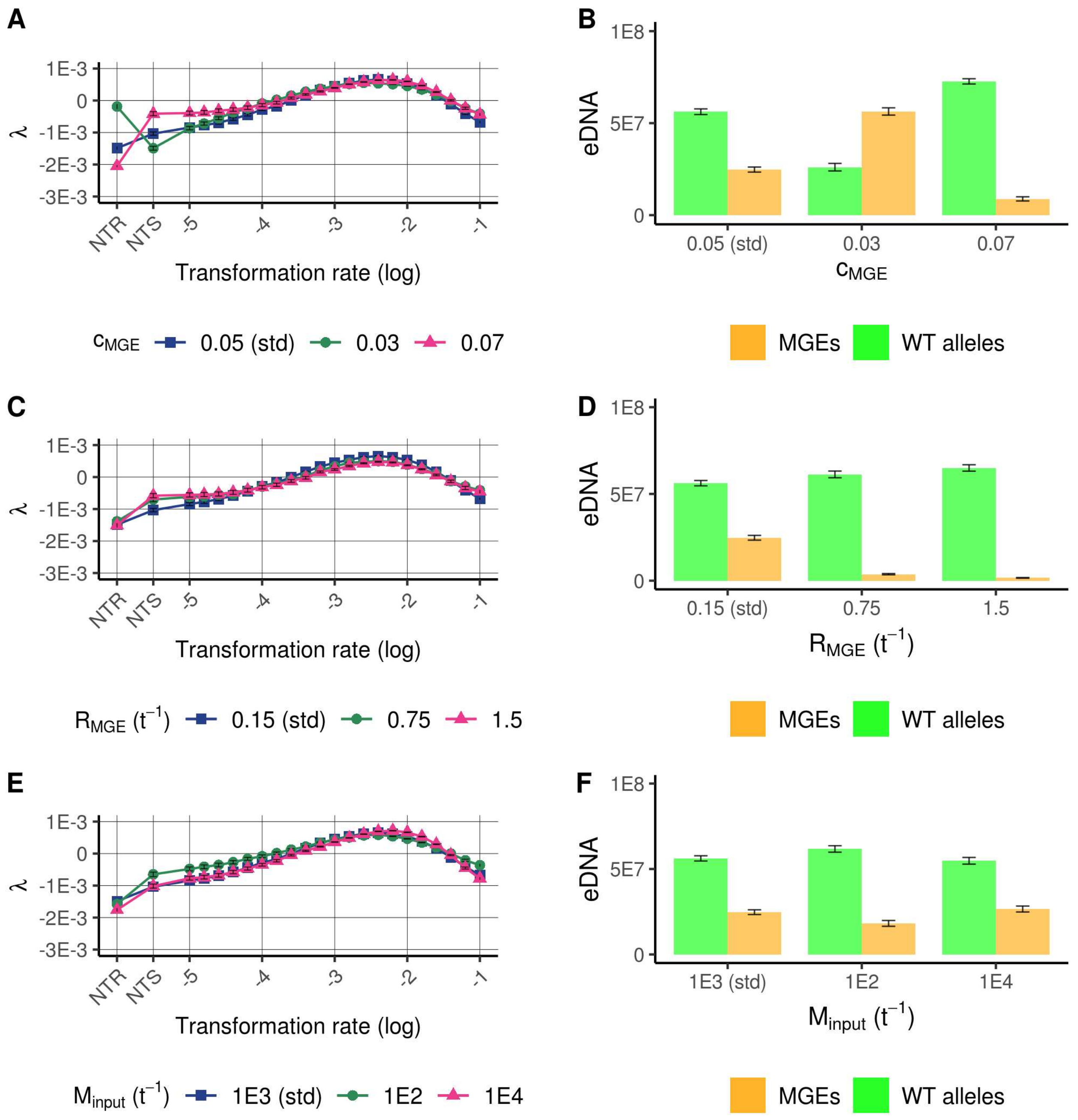

Sup. Figure 6: Sensitivity analysis to model parameters. (A,B) Fitness cost induced by MGEs c_{MGE} (growth reduction of the host). (C,D) Decay rate of extracellular MGEs R_{MGE}, creating an asymmetry with the degradation of extracellular WT alleles which remains at the standard value 0.15. (E,F) Residual input of MGE molecules in the extracellular compartment $M_{input,MGE}$. See Methods in main text for details. λ is the mean stochastic growth rate and eDNA correspond to the mean eDNA molecules at the end of simulations. Error bars are the standard error of 200 simulations. Standard parameters (std) refer to the main text Table 1 and the stress frequency $10^{-3}t^{-1}$.
