# Supplementary material for: Bacterial Transformation Buffers Environmental Fluctuations through the Reversible Integration of Mobile Genetic Elements
Source: mBio. 2020 Mar 3;11(2):e02443-19. doi: 10.1128/mBio.02443-19 (PMC7064763; doi:10.1128/mBio.02443-19)
Supplement: FIG S7 [file mBio.02443-19-sf007.pdf]

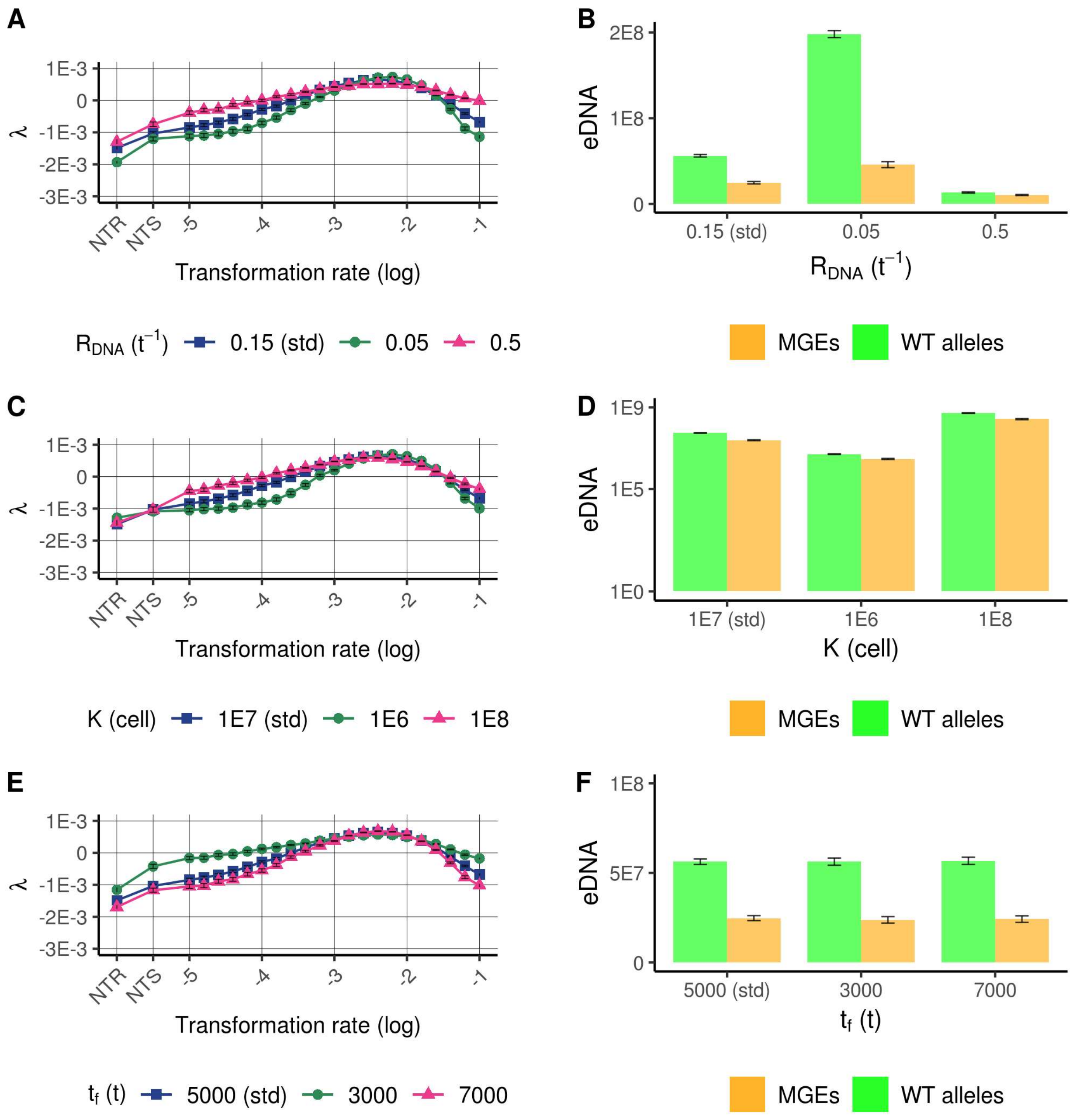

Sup.Figure 7: Sensitivity analysis to model parameters. (A,B) eDNA decay rate R_{DNA} of both extracellular WT alleles and MGEs. (C,D) Carrying capacity of the environment *K*. The size of the initial cell population is *K*/10. (E,F) Simulation time t_f. See Methods in main text for details. λ is the mean stochastic growth rate and eDNA correspond to the mean eDNA molecules at the end of simulations. Error bars are the standard error of 200 simulations. Standard parameters (std) refer to the main text Table 1 and the stress frequency $10^{-3}t^{-1}$.
