# Supplementary material for: Bacterial Transformation Buffers Environmental Fluctuations through the Reversible Integration of Mobile Genetic Elements
Source: mBio. 2020 Mar 3;11(2):e02443-19. doi: 10.1128/mBio.02443-19 (PMC7064763; doi:10.1128/mBio.02443-19)
Supplement: FIG S8 [file mBio.02443-19-sf008.pdf]

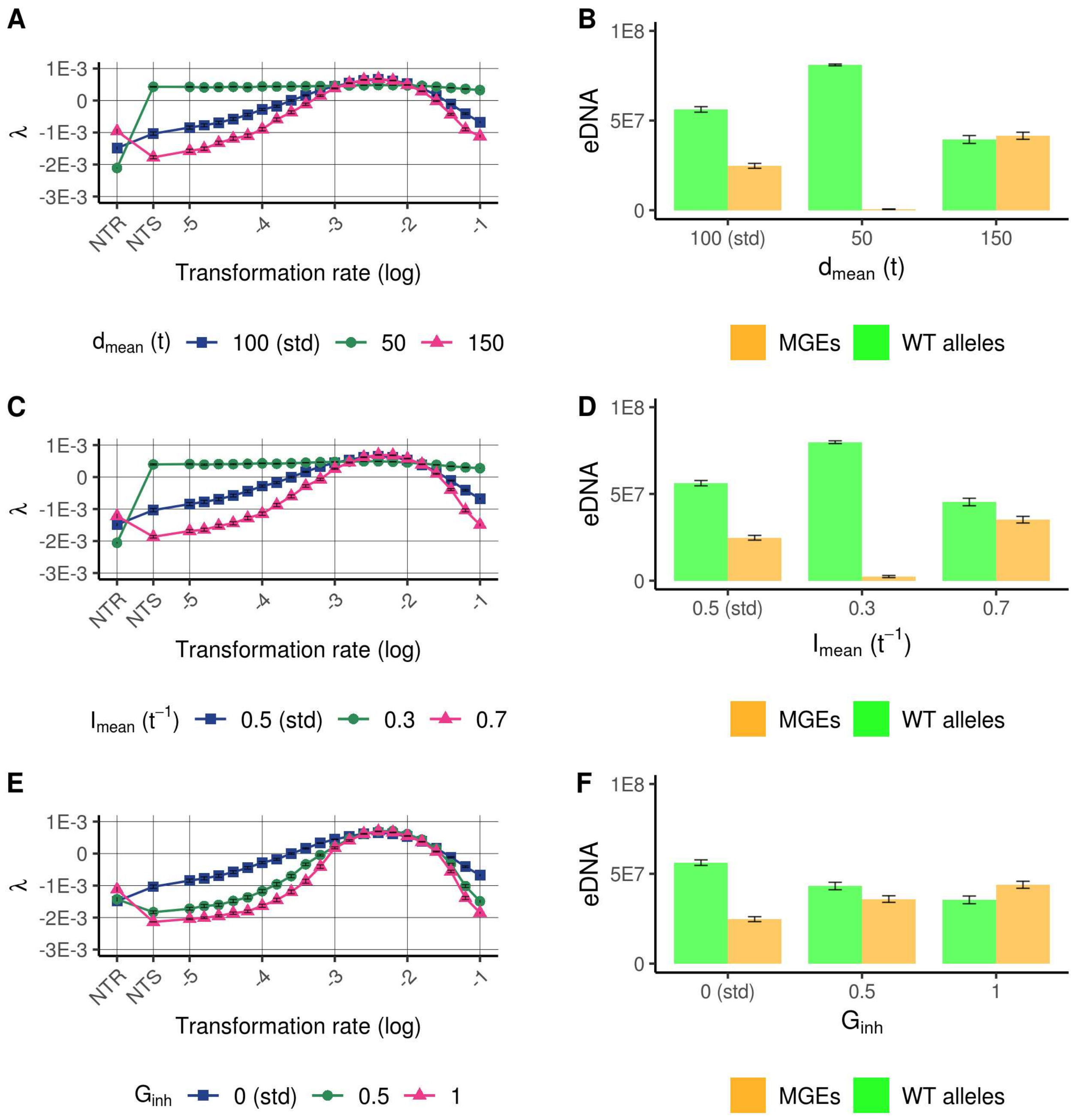

Sup. Figure 8: Sensitivity analysis to model parameters. (A,B) Mean stress duration d_{mean}. (C,D) Mean stress intensity I_{mean}. (E,F) Growth inhibition during stress G_{inh}. When $G_{inh} > 0$, the stress is considered bacteriostatic and reduces growth by the factor $(1 - G_{inh})$ but does not affect lysis rate. See Methods in main text for details. λ is the mean stochastic growth rate and eDNA correspond to the mean eDNA molecules at the end of simulations. Error bars are the standard error of 200 simulations. Standard parameters (std) refer to the main text Table 1 and the stress frequency $10^{-3}t^{-1}$.
